# Synthesis and Characterization of Mn₂O₃ and Its Electrochemical Properties in Relation to Dopamine

**DOI:** 10.7759/cureus.67890

**Published:** 2024-08-27

**Authors:** Sanjana Suresh, Sherin Celshia, Muthamizh Selvamani, Vasugi Suresh, Mohammed Asif Hussein

**Affiliations:** 1 Physiology, Saveetha Dental College and Hospitals, Saveetha Institute of Medical and Technical Science, Saveetha University, Chennai, IND

**Keywords:** nanoparticle, mn2o3, neurotransmitter, biosensor, dopamine

## Abstract

Introduction

Manganese(III) oxide (Mn_2_O_3_) is a transition metal oxide that has gained significant attention due to its unique properties and potential applications in various fields, including catalysis, energy storage, and sensing. Dopamine, a neurotransmitter in the human brain, plays a crucial role in regulating several physiological processes as its detection is important in areas such as medical diagnostics and neurochemistry. The synthesis of Mn_2_O_3_ can be achieved through methods like precipitation, hydrothermal synthesis, or solid-state reactions.

Aims

The objective of this study is to synthesize Mn_2_O_3_, characterize its structure and morphology, and investigate its electrochemical properties toward dopamine.

Materials and methods

Materials used included manganese sulfate (MnSO_4_), potassium permanganate, deionized water, a Teflon steel autoclave, and a hot air oven. For the synthesis of a hierarchical Mn_2_O_3_ rodlike shape, MnSO_4_•H_2_O (8 mmol) and potassium permanganate (8 mmol) were firstly dissolved in deionized water (40 mL) by stirring, which was then transferred to a Teflon-lined stainless steel autoclave (50 mL). This autoclave was sealed and maintained at 90℃ for six hours. Finally, the resultant Mn_2_O_3_ rods were collected by filtration, washed with distilled water and absolute ethanol for several times, and dried in air at 80℃. Mn_2_O_3_ rods were obtained by the calcinations of the as-prepared Mn_2_O_3_ rods at different temperatures. When Mn_2_O_3_ rods were treated at 600℃ for six hours in air, Mn_2_O_3_ rods could be collected.

Results

The X-ray diffraction (XRD) analysis shows that Mn_2_O_3 _is crystalline in structure and it matched with that of the Joint Committee on Powder Diffraction Standards (JCPDS). The field emission scanning electron microscopy (FE-SEM) shows the morphology of Mn_2_O_3 _is a particle with the size of 100 nm. Cyclic voltammetry response shows that compared to bare electrode, modified electrode shows the higher current response which indicates the sensing ability of the dopamine molecule.

Conclusion

Mn₂O₃ was prepared using a hydrothermal technique, and the formation of nanoparticles (NPs) was verified through XRD, while the morphology was examined using FE-SEM. The Mn_2_O_3_ obtained was utilized in the detection of electrochemical dopamine, showing promise in the development of effective dopamine sensors. This study sets the stage for the integration of Mn₂O₃ into microfluidic systems for ongoing dopamine monitoring, presenting novel prospects for healthcare and neurochemical investigations. The exploration of various surface engineering approaches may additionally improve the electrochemical capabilities of Mn₂O₃ for the advancement of sensor technology.

## Introduction

Biosensors are devices designed to detect and respond to particular analytes present in a sample. They employ electrochemical, optical, or other transducers in combination with a biological recognition system to convert the concentration of the analyte into an electrical signal. Such devices find applications in bioprocessing, medical diagnostics, agriculture, and environmental monitoring [1]. In recent years, the performance of electrochemical sensors has been substantially improved by the production of cutting-edge nanomaterials, enabling quick and precise analysis of target analytes. Due to their ease of use, low cost, and high sensitivity, electrochemical sensors have become potential tools for identifying and quantifying pharmacological compounds. Metal oxides have drawn a lot of interest among the many materials investigated for sensing applications because of their special physicochemical characteristics and promise for electrochemical sensing [2]. The environmentally friendly characteristics of metal nanoparticles (NPs) synthesized through biological means have attracted considerable interest in material synthesis. Biologically produced NPs provide better regulation of size distribution in comparison to other techniques. The use of biological processes, including microbes, biomolecules, and plant extracts, for NP production has become an appealing approach due to its numerous advantages over conventional chemical methods [3].

Manganese(III) oxide (Mn_2_O_3_) is a chemical compound formed by the combination of two manganese (Mn) atoms and three oxygen (O) atoms. It is an inorganic substance found in both alpha and beta Mn_2_O_3_ crystal forms, both of which are used in a variety of applications. Studies have investigated the electrochemical application of Mn_2_O_3_ in lithium-ion batteries and other electrochemical devices. Mn is also preferred due to its low cost, low toxicity, and environmentally friendly characteristics [4]. It has been used as black pigments in ceramics, and it is also used as an adsorbent to remove heavy metals and contaminants from water. Mn_2_O_3_ can be synthesized through various methods like the thermal decomposition of Mn precursors, precipitation from Mn salts in the presence of appropriate reducing agents or oxidizing agents, and hydrothermal synthesis [5]. Due to their versatile applications and nontoxic nature, such as electrochemical storage, ion exchange, adsorption, catalysis, and biosensing, nanosized Mn_2_O_3_ and their composites have attracted a lot of attention [6]. Mn_2_O_3_ NPs also has beneficial effects on *Leishmania major* in vitro and in vivo and is used as a potential candidate to treat this infection [7]. Biosensors play a crucial role in detecting specific substances within a sample through the use of electrochemical, optical, or other transducers in combination with a biological recognition system to translate the substance's concentration into an electrical signal. These devices find widespread application in bioprocessing, medical diagnostics, agriculture, and environmental monitoring [8]. Endogenous substances called neurotransmitters enable communication between neurons throughout the body. Through the mechanism of chemical synaptic transmission, they allow the brain to perform a range of operations [9]. The body utilizes various neurotransmitters for different physiological functions, such as acetylcholine, glutamate, gamma-aminobutyric acid (GABA), glycine, dopamine, norepinephrine, and serotonin [10]. Dopamine, a major neurotransmitter plays an important role in many brain functions, and it has also been implicated in neurological disorders [11]. Dopamine belongs to a class of neurotransmitters called catecholamines, and it is synthesized from the amino acid tyrosine [12]. Dopamine as a neurotransmitter has important roles in our cardiovascular and central nervous system, low levels of dopamine in central nervous system indicates several neurological disorders like schizophrenia, Parkinson's disease, Alzheimer’s disease stress, and depression [13]. Elevated dopamine levels play a crucial role in detecting cardiotoxicity, ultimately resulting in heart failure and hypertension [14]. Various methods are employed to measure dopamine levels, including enzyme assays, liquid chromatography, mass spectroscopy, and capillary electrophoresis. For accurate quantitative analysis of dopamine, high-performance liquid chromatography (HPLC) coupled with tandem mass spectrometric (MS/MS) detection is utilized, albeit at a considerable expense [15]. Hydrothermal synthesis is a commonly used method for preparing nanomaterials. In hydrothermal synthesis, nanomaterials can be generated at a number of temperatures, including very high temperatures and room temperature. Hydrothermal synthesis can generate nanomaterials which are not stable at high temperatures; it can also generate nanomaterials with high vapor pressure by allowing minimum loss of material; and the composition of nanomaterial can also be controlled through multiphase or liquid phase reactions [16]. Recently, there has been a lot of interest in the hydrothermal synthesis of metal carbonates and their subsequent thermal conversion into corresponding metal oxide NPs. When this process breaks down thermally, CO2 gas is released, which causes nanopores to form in the final products. These nanopores aid in the creation of novel, highly surface-area nanostructures [17]. Electrochemical sensing is a robust method employed for the identification and measurement of different chemical substances within a liquid medium, relying on the interplay between the analyte and the electrode surface within an electrochemical cell. The fundamental elements of electrochemical sensing encompass the electrode, electrolyte, and electrical cell [18]. The process involves the application of potential or current to the working electrode, which in turn induces a redox reaction at the electrode surface. This reaction depends on the concentration of the analyte. There are two techniques of electrochemical sensing voltammetry and amperometry. Voltammetry: The working electrode's potential is applied, and the resulting current is measured as the potential is scanned linearly or sequentially. The resulting voltammogram offers information on the analyte's redox behavior and can be utilized to determine its concentration. Electrochemical sensing is used in various fields due to its advantages which include high sensitivity, selectivity, and the ability to perform real-time measurements in a cost-effective manner [19].

In this study, we have synthesized and characterized Mn_2_O_3_ crystals and investigated their electrochemical property toward dopamine sensing using hydrothermal methods.

## Materials and methods

Materials

MnSO_4_, potassium permanganate, and dopamine were purchased from Sigma-Aldrich. Deionized water, ethanol, Teflon steel autoclave (The Chemours Company, Wilmington, USA), magnetic stirrer, muffle furnace, beaker, glassy carbon electrode (GCE), counter electrode (platinum wire), reference electrode (Ag/AgCl), and pipette were also used in the study.

Methods

Synthesis of Mn_2_O_3_ NP 

MnSO_4_•H_2_O (8 mmol) and potassium permanganate (8 mmol) were first mixed in deionized water (40 mL) by stirring, and then the resultant solution was transferred to a Teflon-lined stainless steel autoclave (50 mL) for the production of hierarchical Mn_2_O_3_ microspheres. The autoclave was sealed and kept at 90°C for six hours. The resultant Mn_2_O_3_ was then filtered, repeatedly rinsed in distilled water and 100% ethanol, and allowed to dry in the air at 80°C. Mn_2_O_3_ microspheres were created by calcining the Mn_2_O_3_ microspheres as prepared at various temperatures. Mn_2_O_3_ microspheres have been collected after being exposed to 600°C for six hours in air. The process of synthesizing the required Mn_2_O_3 _nanoflower through the hydrothermal technique has been depicted in Figure ​1. 

**Figure 1:**
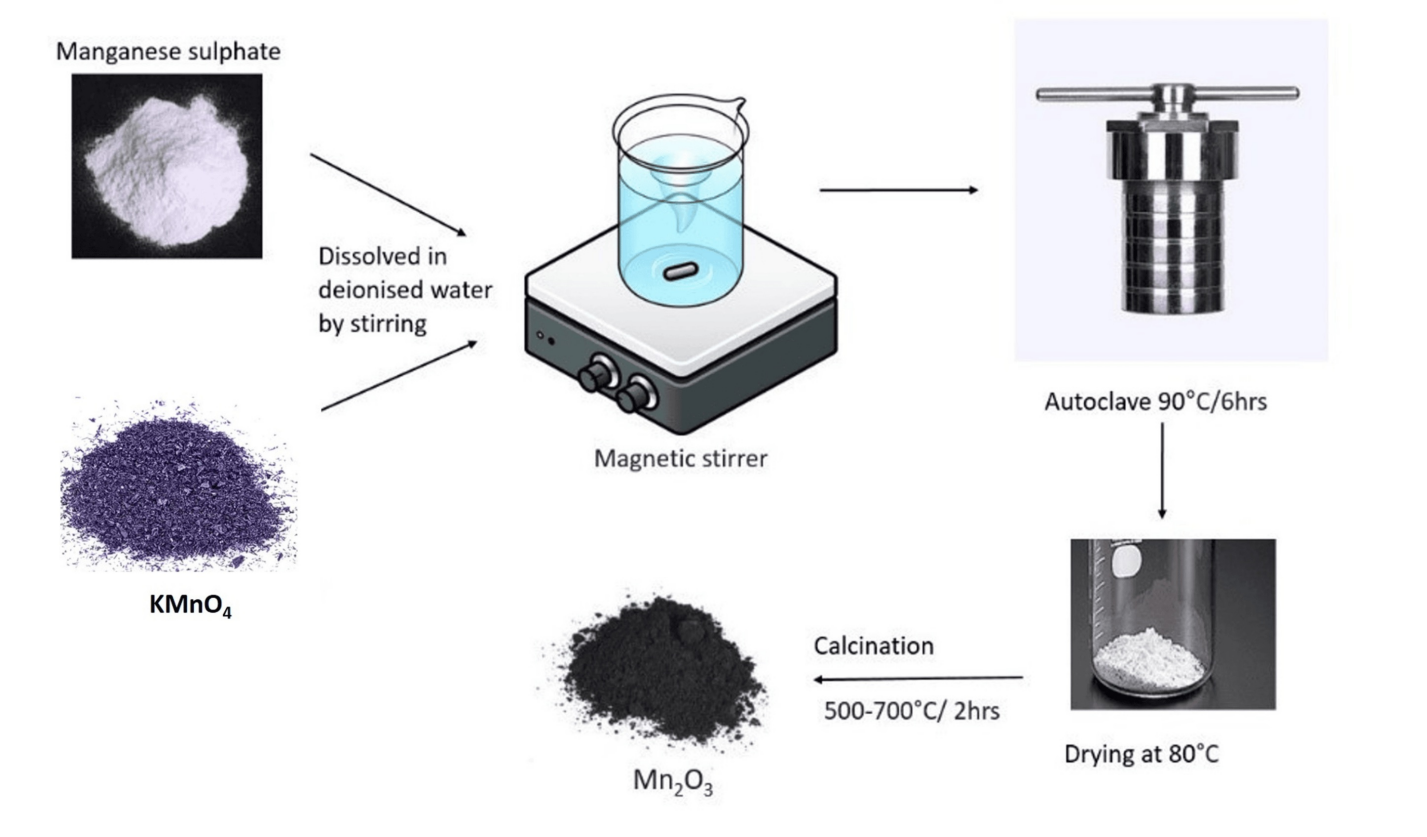
The process of synthesizing of the Mn2O3 through the hydrothermal technique Mn_2_O_3_: Manganese(III) oxide

Electrode Preparation Procedure

The GCE was modified with Mn_2_O_3_ before that working electrode was mechanically polished with 1 µm, 0.3 µm, and 0.05 µm alumina pastes for mirror finishing. Then, it was subjected to ultrasonication in double distilled water for a few minutes to clean the surface of GCE. The Mn_2_O_3_ save suspension was prepared by dispersing 5 mg of Mn_2_O_3_ in 10 mL of ethanol during 20 minutes of ultrasonic agitation. Subsequently, the GCE was coated with 10 µL of the suspension using the drop coating method and then dried in air. The fabricated working electrode was used for the sensing of dopamine by electrochemical method.

## Results

X-ray diffraction (XRD) analysis

**Figure 2:**
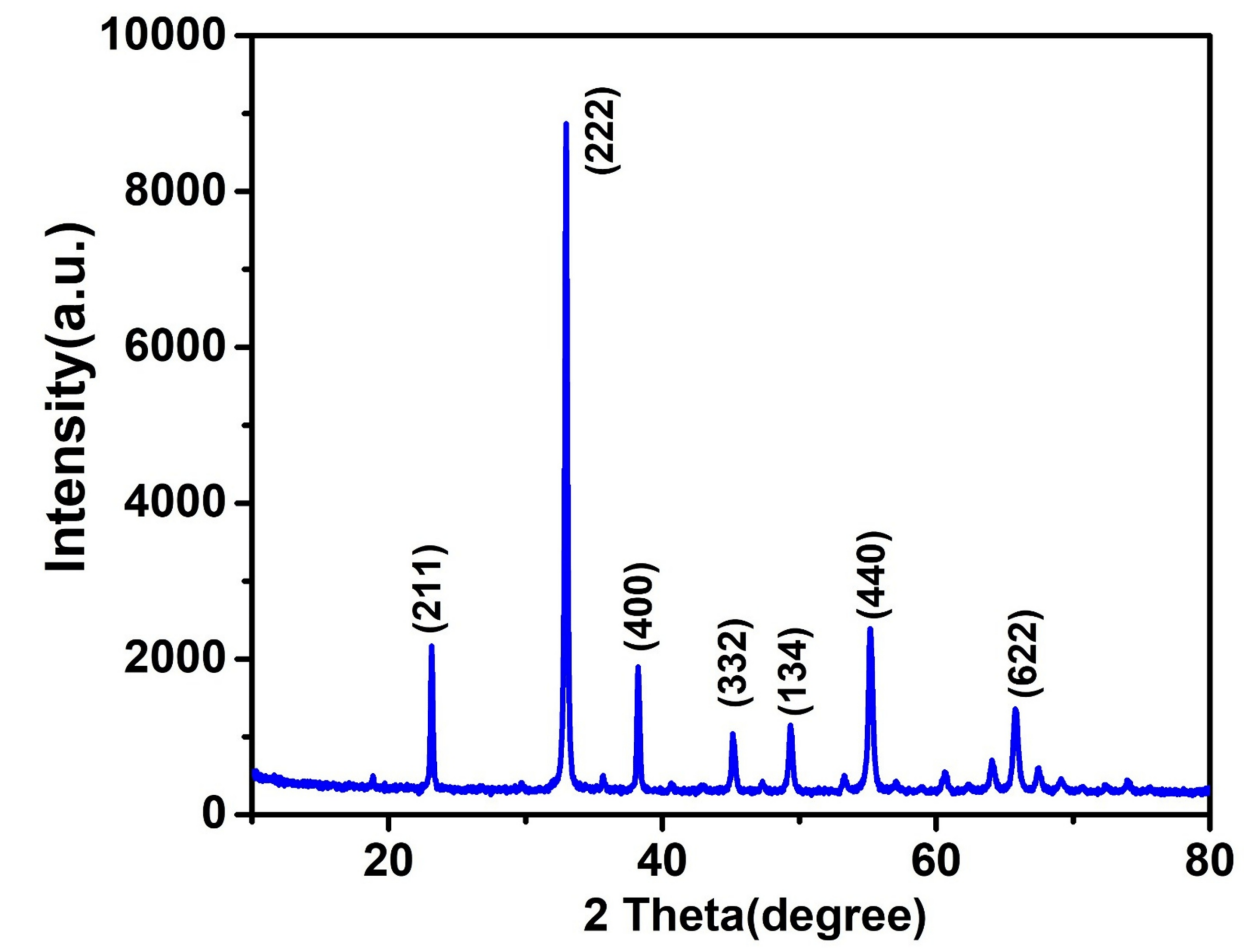
An XRD pattern of Mn₂O₃ nanoparticle XRD: X-ray diffraction; Mn₂O₃: manganese (III) oxide

The crystalline features and phase purity of the synthesized nanomaterial Mn_2_O_3_ were investigated using XRD patterns as shown in Figure 2. The sharp diffraction peaks were observed at 33.09 (222), 38.31 (400), 55.38 (440), 64.23 (541), and 65.92 (622). The XRD pattern was confirmed with the reference pattern of Mn_2_O_3_ (JCPDS 41-144) [20]. Further, no impurity-related visible diffraction peaks were identified, confirming the high purity of Mn_2_O_3_ . 

Morphological analysis

**Figure 3:**
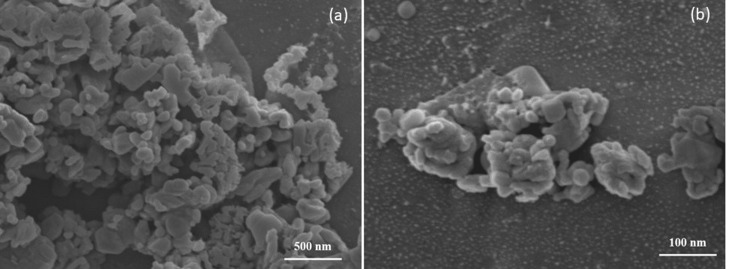
The FE-SEM images of (a and b) represent Mn2O3 nanoparticle prepared by hydrothermal treatment FE-SEM: Field emission scanning electron microscopy; Mn_2_O_3_: manganese (III) oxide (a) Mn_2_O_3_ at 500 nm magnification; (b)  Mn_2_O_3_ at 100 nm magnification

**Figure 4:**
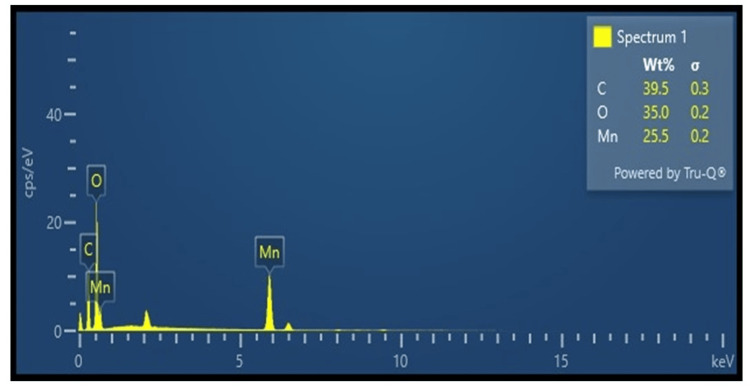
The EDX analysis result of the Mn203 particles synthesized EDX: Energy-dispersive X-ray; Mn_2_0_3_: manganese (III) oxide

Field emission scanning electron microscopy (FE-SEM) is used for determining the surface morphologies and microstructures of the nanomaterial Mn_2_O_3_. Figure 3 shows the FE-SEM analysis of NP Mn_2_O_3_; the image reveals that the particle is rodlike shaped with an overall particle size of 100 nm, and it was distributed evenly. Figure 4 indicates the EDX analysis; the elemental composition of synthesized Mn_2_O_3_ included 39.5% of carbon, 35.0% of oxygen, and 25.5% of manganese. The EDX analysis showed the presence of impurities like carbon and oxygen in the synthesized Mn_2_O_3_ NP. 

Cyclic voltammetry

**Figure 5:**
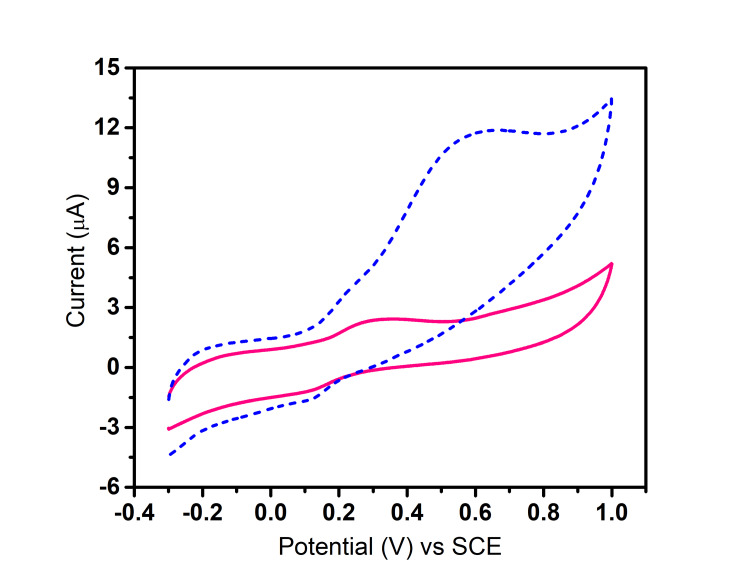
Cyclic voltammetric response of bare and Mn203-modified GCE toward dopamine at an applied potential of 50 mV/s GCE: Glassy carbon electrode; SCE: saturated calomel electrode The pink line indicates bare GCE electrode response toward dopamine sensing. The blue line indicates Mn_2_0_3_-modified GCE response toward dopamine sensing

Figure 5 shows the cyclic voltammetric response of a modified electrode at various potentials; the Mn_2_O_3_-modified electrode showed distinct redox peaks in the presence of dopamine. The anodic peak was around +0.6 V and the cathodic peak at +0.3 V corresponding to the oxidation and reduction potentials, respectively. The electrochemical behavior of the redox peaks indicated effective electron transport between the electrode and dopamine molecules. Cyclic voltammetry response showed that compared to bare electrode, modified electrode shows higher current response which indicates sensing ability of Mn_2_O_3 _NPs. 

**Figure 6:**
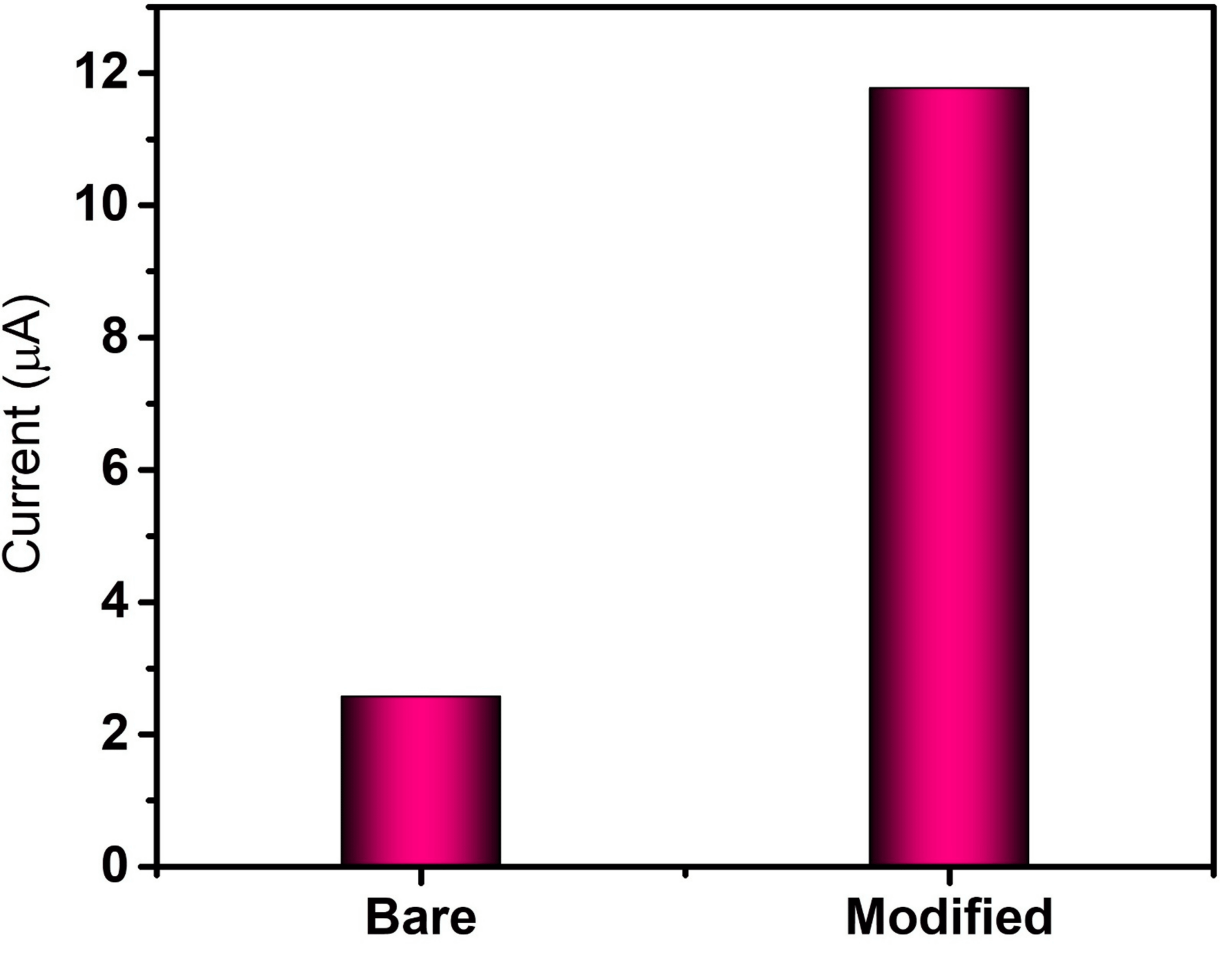
Cyclic voltammetry current response of bare and Mn2O3-modified electrode toward dopamine GCE: Glassy carbon electrode

In Figure 6, for the bare electrode, a potential of 50 mV/s resulted in a corresponding current response of 2.5 μA, while for the electrode modified with Mn_2_O_3_, the application of a potential of 50 mV/s led to a corresponding current response of 11.7 μA. The increased current response denotes the increased sensing ability of Mn_2_o_3_ NP toward dopamine. 

 Scan rate 

**Figure 7:**
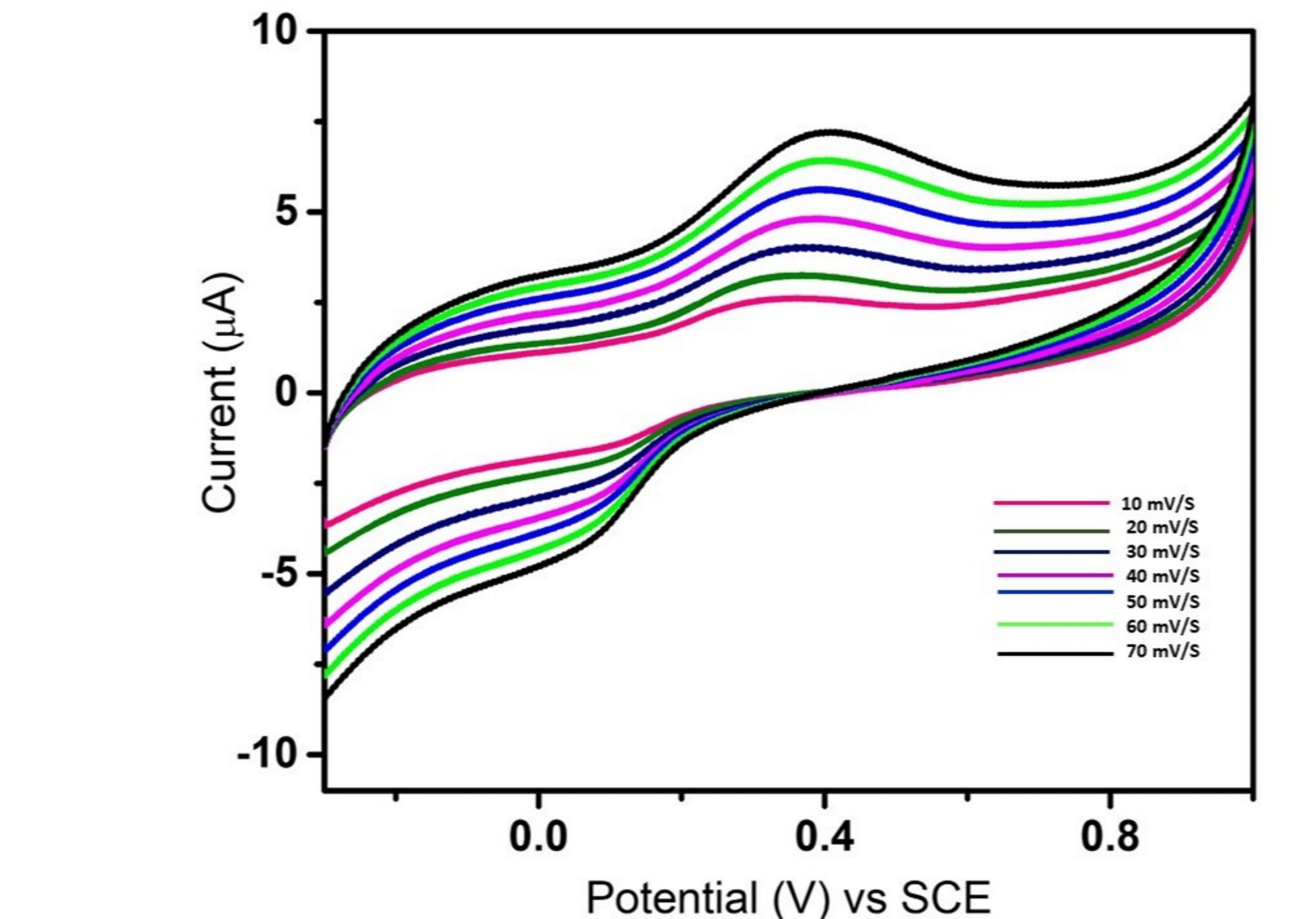
Scan rate effect of Mn2O3 modified GCE toward dopamine 10-70 mV/s GCE: Glassy carbon electrode; SCE: saturated calomel electrode

The process of scanning includes gradually varying the voltage, usually between 10 and 70 mV/s; this results in various modifications to the cyclic voltammogram that is produced. Figure 7 shows the scan rate result for Mn_2_O_3 _NP synthesis. While applying potentials ranging from 10 mV/s to 70 mV/s in increments of 10 mV/s each time, the current response readings were recorded as shown in Figure 7. As can be seen from the plotted graphs and results, there was a consecutive increase in the current response in line with the application of rising potentials one after the other. These findings confirm the high stability and sensitivity of Mn_2_O_3_ NPs toward sensing of dopamine.

## Discussion

Mn_2_O_3 _can be used in a variety of ways because of its intriguing electrochemical characteristics. It can be used as an electrode material in batteries and supercapacitors, among other electrochemical cells. Mn_2_O_3_ can store a sizable amount of charge due to its high specific capacitance. Moreover, it has strong electrochemical stability, enabling repeated cycling without noticeably degrading the material. Furthermore, Mn_2_O_3_ has the ability to catalyze a number of electrochemical processes, such as dopamine oxidation. Because it may be used as an electrode material to detect and measure dopamine levels in biological samples, this characteristic makes it relevant for dopamine research [21]. Mn_2_O_3 _NPs prepared by a noninvasive electromagnetic field were used for treating Parkinson’s disease [22]. Studies have shown that a highly stable and sensitive graphic oxide-1,4-xylenediamine-coated Mn_2_O_3_ particle was used for simultaneous electrooxidation of paracetamol and ascorbic acid [23].

Figure 2 displays an XRD pattern of the Mn_2_O_3_ NP that was generated electrochemically following a hydrothermal treatment. Through XRD examination, the properties and makeup of the synthesized Mn_2_O_3_ chemical were verified. The outcome shows that there are no peaks that relate to any other compounds save Mn_2_O_3_. After that, the acquired peaks were compared to those of the standard JCPDS no. 41-144, and they were discovered to correspond to Miller indices of (222), (400), (440), (541), and (622) and have been observed to exhibit peaks. The tall peaks found in the analysis indicate the highly crystalline structure of the Mn_2_O_3_ molecule that was synthesized. A study showed that Mn_2_O_3_ NP showed well-defined diffraction peaks as (200), (211), (222), (400), (332), (422), (431), (440), and (622). The investigation revealed that the Mn_2_O_3_-titanium dioxide (TiO_2_)-decorated graphene, synthesized through a single-step sol-gel process, proved to be efficient in the rapid and selective ultrasensitive electrochemical detection of dopamine. The pristine graphene and graphene oxide were decorated with TiO_2_ and Mn_2_O_3_ for the purpose of dopamine sensing. The resulting binary and ternary composites were subjected to characterization, with the ternary composite exhibiting higher electrochemical activities in comparison to the binary composite when exposed to the potassium hexacyanoferrate redox probe. The ternary composite was further validated for dopamine sensing by introducing commercial dopamine into blood serum and urine samples, resulting in a 98% signal recovery. The developed sensor demonstrated favorable stability and reproducibility [24]. Another study showed that Mn_2_O_3_ powder composed of porous double-shelled hollow microspheres can be synthesized by a multistage procedure involving precipitation and controlled oxidation [25]. Research has demonstrated the imitative behavior of a Mn (II) compound, in which MnO NPs anchored on porous carbon (MnO NPs/PC) were deliberately synthesized through the in situ transformation of MnO_2_ nanobelts supported on porous carbon [26]. A study indicates that Mn_2_O_3_-doped MnO exhibits superior catalytic performance and stronger stability when it comes to oxygen reduction when compared to pure MnO. Specifically, the cyclic voltammetry results demonstrate that the onset potential of oxygen reduction with the catalysis of Mn_2_O_3_ doped MnO was -0.2V and 0.3V [27]. In the aforementioned study, it was demonstrated that the incorporation of gold and carbon nanomaterials with MIPs can enhance the conductivity of sensing materials. Specifically, a dopamine-imprinted sensor was developed by depositing gold NPs onto a glassy carbon electrode, subsequently forming a self-assembled monolayer of p-thioaniline on the electrode surface through the interaction of Au-S bonds between gold NPs and thiol groups [28].

Limitation

Achieving pure Mn₂O₃ and controlling its particle size and morphology present significant challenges, which can influence the consistency of its electrochemical performance. While advanced characterization methods like XRD, scanning electron microscopy (SEM), and Brunauer-Emmett-Teller (BET) surface area analysis are crucial, they have inherent limitations in resolution and precision. The stability of Mn₂O₃ during electrochemical testing is also a concern, as structural changes may impact its effectiveness. Moreover, slight variations in synthesis and testing conditions can lead to reproducibility issues. Additionally, electrochemical techniques might struggle with detecting low dopamine concentrations, which can affect the sensitivity and accuracy of the results.

## Conclusions

Mn_2_O_3_ was synthesized by hydrothermal method, the formation of NP was confirmed by XRD analysis, morphology was confirmed by FE-SEM analysis, and the synthesized nanomaterial was used for the electrochemical sensing of dopamine. The synthesis of Mn_2_O_3_ paves the way for more efficient dopamine sensing platforms, and the integration of this NP in microfluidic devices enables continuous monitoring of dopamine levels which opens new possibilities for healthcare and neurochemical research. Investigating the effects of different surface engineering strategies on the electrochemical performance of Mn_2_O_3 _can lead to the development of more advanced dopamine sensors.

## References

[1] Thalir S, Celshia Susai S, Selvamani M, Suresh V, Sethuraman S, Ramalingam K (2024). Sensing of quercetin with cobalt-doped manganese nanosystems by electrochemical method. Cureus.

[2] Krisha SG, Menaka S, Celshia S, Selvamani M, Suresh V (2024). Synthesis of copper molybdate and its electrochemical sensing of paracetamol. Cureus.

[3] S K, A G, I G K I, S V, P S, S B (2024). Facile synthesis of silver nanoparticles from sustainable Sargassum sp. seaweed material and its anti-inflammatory application. Cureus.

[4] Ridgway RH (1933). Manganese: General Information. June 1933.

[5] Kaladi Chondath S, Menamparambath MM (2023). Self-assembly of random networks of zirconium-doped manganese oxide nanoribbons and poly(3,4-ethylenedioxythiophene) flakes at the water/chloroform interface. Faraday Discuss.

[6] Mohamed Abdel Salam (2015). Synthesis and characterization of novel manganese oxide nanocorals and their application for the removal of methylene blue from aqueous solution. Chem Eng J.

[7] Tavakoli P, Ghaffarifar F, Delavari H, Shahpari N (2019). Efficacy of manganese oxide (Mn(2)O(3)) nanoparticles against Leishmania major in vitro and in vivo. J Trace Elem Med Biol.

[8] Mathew MZ, Celshia S, Selvamani M, Suresh V, Hussein MA (2024). The synthesis of FeS and investigation on electrochemical sensing toward neuroprotector. Cureus.

[9] Rizo J (2018). Mechanism of neurotransmitter release coming into focus. Protein Sci.

[10] Gray EG, Gordon-Weeks PR, Burgoyne RD (2013). Neurotransmitter interaction and compartmentation. https://books.google.com.ph/books?hl=en&lr=&id=r6jaBwAAQBAJ&oi=fnd&pg=PA2&dq=Neurotransmitter+Interaction+and+Compartmentation.+Springer+Science+%26+Business+Media&ots=La9VIspSAt&sig=SggPgewXzosC-jh5h6cHXEX3iEA&redir_esc=y#v=onepage&q=Neurotransmitter%20Interaction%20and%20Compartmentation.%20Springer%20Science%20%26%20Business%20Media&f=false.

[11] Ko JH, Strafella AP (2012). Dopaminergic neurotransmission in the human brain: new lessons from perturbation and imaging. Neuroscientist.

[12] Siafis S, Wu H, Wang D (2023). Antipsychotic dose, dopamine D2 receptor occupancy and extrapyramidal side-effects: a systematic review and dose-response meta-analysis. Mol Psychiatry.

[13] Klein MO, Battagello DS, Cardoso AR, Hauser DN, Bittencourt JC, Correa RG (2019). Dopamine: functions, signaling, and association with neurological diseases. Cell Mol Neurobiol.

[14] Bucolo C, Leggio GM, Drago F, Salomone S (2019). Dopamine outside the brain: the eye, cardiovascular system and endocrine pancreas. Pharmacol Ther.

[15] Pérez-Fernández V, Harman DG, Morley JW, Cameron MA (2017). Optimized method to quantify dopamine turnover in the mammalian retina. Anal Chem.

[16] Thakur A, Thakur P, Paul Khurana SM (2022). Synthesis and Applications of Nanoparticles. Springer Nature.

[17] Tao Y, Zhaohui H, Yangai L, Minghao F, Xin O, Meiling H (2014). Controlled synthesis of porous FeCO3 microspheres and the conversion to α-Fe2O3 with unconventional morphology. Ceram Int.

[18] Khaleque MA, Hossain MI, Ali MR, Bacchu MS, Saad Aly MA, Khan MZ (2023). Nanostructured wearable electrochemical and biosensor towards healthcare management: a review. RSC Adv.

[19] Jackowska K, Krysinski P (2013). New trends in the electrochemical sensing of dopamine. Anal Bioanal Chem.

[20] Quanguo H, Jun L, Xiaopeng L (2019). A promising sensing platform toward dopamine using MnO2 nanowires/electro-reduced graphene oxide composites. Electrochimica Acta.

[21] Wise RA, Robble MA (2020). Dopamine and addiction. Annu Rev Psychol.

[22] Wang X, Zhao J, Wang W (2022). Electromagnetic field-enhanced chiral dimanganese trioxide nanoparticles mitigate Parkinson’s disease. Sci China Chem.

[23] Ara E, Seungwon J (2017). A highly stable and sensitive GO-XDA-Mn2O3 electrochemical sensor for simultaneous electrooxidation of paracetamol and ascorbic acid. Electrochim Acta.

[24] Arockiajawahar AG, Karutha PD, Venkataraman D, Jong HH (2019). Single step sol-gel synthesized Mn2O3-TiO2 decorated graphene for the rapid and selective ultra sensitive electrochemical sensing of dopamine. Electrochim Acta.

[25] Qiao Y, Yu Y, Jin Y, Guan YB, Chen CH (2014). Synthesis and electrochemical properties of porous double-shelled Mn2O3 hollow microspheres as a superior anode material for lithium ion batteries. Electrochim Acta.

[26] Jiangyu S, Shiya F, Liu H (2024). Controllable synthesis of MnO nanoparticles supported on porous carbon as a highly active oxidase mimic for dopamine detection. J Alloys Compd.

[27] Lawlor V (2016). Mn2O3 doping induced the improvement of catalytic performance for oxygen reduction of MnO. Int J Hydrogen Energy.

[28] Lakard S, Pavel IA, Lakard B (2021). Electrochemical biosensing of dopamine neurotransmitter: a review. Biosensors (Basel).

